# „If Hormones Make the Man, What Makes the Woman?“: Zu den Anfängen der endokrinologischen Geschlechtsentwicklungsforschung 1900–1950

**DOI:** 10.1007/s00048-026-00439-7

**Published:** 2026-01-28

**Authors:** Xenia Steinbach

**Affiliations:** Institut für Ethik, Geschichte und Philosophie der Medizin, Medizinische Hochschule Hannover (MHH), Carl-Neuberg-Str. 1, 30625 Hannover, Deutschland

**Keywords:** Wissenschaftsgeschichte, Embryonale Geschlechtsentwicklung, Hormone, Experimentalsysteme, Feministische STS, History of science, Embryonic sex development, Hormones, Experimental systems, Feminist STS

## Abstract

Während die genetisch und hormonell gesteuerte pränatale Ausbildung männlicher Geschlechtsorgane seit Beginn des 20. Jahrhunderts zunehmend detailliert beschrieben wurde und inzwischen auch für die ovarielle Entwicklung mehrere beteiligte Gene identifiziert sind, bleiben die hormonellen Faktoren der weiblichen Geschlechtsdifferenzierung bis in die Gegenwart weitgehend unerforscht. Das bis heute leitende physiologische Modell folgt einer binären Logik der An- und Abwesenheit hormoneller sowie genetischer Faktoren: Männlichkeit erscheint darin als Resultat aktiver biologischer Prozesse, Weiblichkeit hingegen als deren Ausbleiben und passive „Standardeinstellung“. Historisch wird diese Modellbildung allem voran auf die tierexperimentellen Arbeiten des französischen Embryologen und Endokrinologen Alfred Jost aus den 1940er Jahren zurückgeführt. Feministische Wissenschaftskritik, die teilweise auch aus den Biowissenschaften selbst kommt, problematisiert bereits seit mehreren Jahrzehnten die darin fortwirkende Dichotomie von Aktivität und Passivität als Reproduktion tradierter kultureller Vorannahmen über Geschlecht sowie das aus ihrer Sicht erklärungsbedürftige Desiderat der Erforschung der weiblichen Geschlechtsdifferenzierung. Der vorliegende Beitrag knüpft an diese Kritik an und erweitert sie um eine historische Analyse der materiell-technischen Bedingungen der frühen embryonalen Geschlechtsentwicklungsforschung in der ersten Hälfte des 20. Jahrhunderts. Anhand von vier zentralen Experimentalsystemen – (1) den anatomisch-morphologischen Untersuchungen von Pol Bouin und Paul Ancel um 1900, (2) der Freemartin-Forschung der 1910er Jahre, (3) den Geschlechtsumwandlungsexperimenten von Eugen Steinach in den 1910er Jahren sowie (4) der embryologischen Sexualhormonforschung von Vera Dantschakoff in den 1930er Jahren – wird aufgezeigt, wie sich das binäre, auf Männlichkeit konzentrierte Modell der embryonalen Geschlechtsentwicklung bereits vor den 1940er Jahren unter dem Einfluss spezifischer materieller und technischer Forschungsbedingungen der frühen endokrinologischen Experimentalsysteme herausbildete. Ziel ist es, die Rolle dieser materiell-technischen Bedingungen und insbesondere von Hormonen als epistemische, technische und teils widerspenstige Dinge innerhalb dieser Systeme sichtbar zu machen und mögliche Pfadabhängigkeiten bis in gegenwärtige experimentelle Konfigurationen der Geschlechtsentwicklungsforschung zu diskutieren.

## Abstract (en)

While the genetically and hormonally controlled prenatal development of male genital organs has been described in increasing detail since the beginning of the 20th century, and several genes involved in ovarian development have now been identified, the hormonal factors involved in female genital differentiation remain largely unexplored to the present day. The physiological model that continues to prevail today follows a binary logic of the presence and absence of hormonal and genetic factors: masculinity appears as the result of active biological processes, while femininity appears as their absence and passive “default pathway”. Historically, this model can be traced back primarily to the animal experiments conducted by the French embryologist and endocrinologist Alfred Jost in the 1940s. Feminist criticism of science, some of which comes from the life sciences themselves, has for several decades been problematising the continuing dichotomy of activity and passivity as a reproduction of traditional cultural assumptions about gender, as well as the desideratum of researching prenatal female sex differentiation, which in their view requires explanation. This article takes up this longstanding critique and expands on it with a historical analysis of the material and technical conditions of early embryonic sex development research in the first half of the 20th century. Based on four central experimental systems—(1) the anatomical and morphological studies of Pol Bouin and Paul Ancel around 1900, (2) research on Freemartinism in the 1910s, (3) Eugen Steinach’s sex reversal experiments in the 1910s, and (4) Vera Dantschakoff’s embryological sex hormone research in the 1930s—it is shown how the binary, masculinity-focused model of embryonic sex development was already taking shape before the 1940s under the influence of specific material and technical research conditions of the early endocrinological experimental systems. The aim is to highlight the role of these material and technical conditions, and in particular of hormones as epistemic, technical and sometimes rebellious things within these systems, and to discuss possible path dependencies up to current experimental configurations in sex development research.

## __________________________________________________________________

Wissenschaftliche Geschichten^1^ über die menschliche embryonale Geschlechtsentwicklung sind Erzählungen von An- und Abwesenheiten (Richardson 2022: 8). Männliche Geschlechtsmerkmale, so lautet das zentrale biologische Paradigma der pränatalen Geschlechtsdifferenzierung seit Mitte des 20. Jahrhunderts, resultieren aus der Einwirkung „männlicher“ Hormone – der sogenannten Androgene^2^ – in frühen embryonalen Entwicklungsphasen, weibliche Geschlechtsmerkmale entstehen hingegen bei deren Fehlen (Persaud & Torchia 2026: 298). Die weibliche Geschlechtsentwicklung geschieht in dieser binären Logik quasi automatisch als eine Art „Standardeinstellung“ des Körpers (Holterhus & Hiort 2021: 371). Dieses sogenannte „Prinzip der primären Weiblichkeit“ (Neumann 1997: 10) wurde in den 1980er Jahren in neueren molekularbiologischen Theorien über die genetischen Faktoren der Geschlechtsdifferenzierung aktualisiert (Richardson 2015: 129). Erneut sollte die An- oder Abwesenheit eines die männliche Entwicklung steuernden Gens, das auf dem „männlichen“ Y‑Chromosom lokalisiert wurde, darüber entscheiden, ob die Geschlechtsentwicklung in die männliche oder weibliche Richtung verläuft (Page et al. 1987: 1099). Geschlechtsausprägungen, die sich im Spektrum zwischen den beiden Polen weiblich und männlich bewegten, wurden dabei stets als Störungen der „normalen“ oder auch „gesunden“ Geschlechtsentwicklung verstanden und als pathologisch markiert.^3^ Während die Kenntnisse über die männliche pränatale Geschlechtsdifferenzierung stetig angewachsen sind, blieben die Faktoren der weiblichen Differenzierungsprozesse bis in die 2000er Jahre noch weitgehend unerforscht und rücken erst allmählich in den Fokus der molekularbiologischen Geschlechtsentwicklungsforschung (Hornig et al. 2023; Hughes 2004; Baskin et al. 2021).

Diese Hinwendung zur weiblichen Geschlechtsdifferenzierung kann auch als Verdienst der feministisch-informierten Wissenschaftsforschung betrachtet werden, die bereits seit über vier Jahrzehnten Kritik am Androgen-Zentrismus der embryonalen Geschlechtsentwicklungsforschung übt (Fausto-Sterling 1989, 2000, 2012; Germon 2014; Richardson 2008; van den Wijngaard 1997). Solche Arbeiten betrachteten unter anderem die sprachliche Theorienbildung in diesem Bereich und kritisierten die Akzeptanz der „Repräsentation von Männlichkeit als Anwesenheit und Weiblichkeit als Abwesenheit“ (Fausto-Sterling 1989: 326) in biologischen Diskursen unter Verweis auf die forschungsstrukturierende Wirkmacht dieser Metapher, deren Ursprung sie allem voran in den Arbeiten des französischen Embryologen und Endokrinologen Alfred Jost Ende der 1940er Jahre sahen (Bernabé 2020; Fausto-Sterling 2000: 199–205; Jordan-Young 2010: 26–29; van den Wijngaard 1997: 29). „Das wissenschaftliche Modell der sexuellen Entwicklung, das sich gegen andere durchgesetzt hat, ist dasjenige, das sich am meisten an solche konservativen Vorstellungen anlehnt, […] die Weiblichkeit durch Passivität und Mangel charakterisieren“, schreibt Anne Fausto-Sterling im Jahr 2000 (204). In ihrer Monografie *Sexing the Body* kritisiert sie damit zum einen Alfred Josts Übernahme problematischer, kulturell tradierter Vorstellungen von Weiblichkeit und Männlichkeit bei der Interpretation seiner Forschungsergebnisse, deren Essenzialisierung er mit der hormonellen Abwesenheitshypothese vorangetrieben habe (Fausto-Sterling 2000: 204). Zum anderen verweist sie auf das aus ihrer Sicht erklärungsbedürftige Desiderat der Erforschung der weiblichen pränatalen Geschlechtsentwicklung: „If Hormones Make the Man, What Makes the Woman?“ war eine Frage, die laut Fausto-Sterling (ebd.: 197) angesichts der Konzeption der embryonalen weiblichen Geschlechtsdifferenzierung als hormonunabhängiger Prozess und voreingestellter „Naturzustand“ aus Sicht der männlich dominierten Forschung der Nachkriegszeit scheinbar keiner weiteren wissenschaftlichen Erklärung bedurfte (ebd.: 203).

Die genannten feministisch-informierten Arbeiten bewegen sich in einem weiteren Feld der Naturwissenschaftskritik und der historischen Geschlechterforschung. Autor:innen aus diesem Feld stellen das Ideal objektiver, wertfreier Forschung grundlegend infrage und verweisen auf die tiefe Verwobenheit von wissenschaftlichen Konzepten zur „Natur der Geschlechter“ mit heteronormativen kulturellen Vorannahmen (Ebeling & Schmitz 2006; Haraway 1989; Hubbard 1990; Koreuber & Aßmann 2018; Roberts 2007; Stammberger 2022). Sie problematisieren den in einer über Jahrhunderte männlich dominierten Wissenschaft tief verwurzelten Androzentrismus sowie die Hegemonialität binärer Geschlechtermodelle in medizinisch-biologischen Diskursen des 19. und 20. Jahrhunderts, denen immer auch Modelle des geschlechtlichen Kontinuums gegenübergestanden haben und auch weiterhin stehen (Fausto-Sterling 1993; Klöppel 2010; 2015; Stoff 2024; Voß 2018).

Der vorliegende Artikel knüpft an diese wichtigen und im Kontext politischer Debatten um Geschlecht entstandenen Arbeiten an und erweitert sie um eine systematische Analyse der materiellen und technischen Bedingungen der frühen Experimentalsysteme der embryologischen Geschlechtsentwicklungsforschung. Die forschungsleitende These ist dabei, dass der Androgen-Zentrismus und der Mangel an Wissen über Mechanismen der weiblichen pränatalen Geschlechtsdifferenzierung in der heutigen Geschlechtsentwicklungsforschung nicht erst mit den Arbeiten von Alfred Jost Ende der 1940er Jahre und im Rahmen der daran anknüpfenden androzentrisch geprägten Forschung der zweiten Hälfte des 20. Jahrhunderts begründet wurde. Vielmehr bahnte er sich bereits in der ersten Hälfte des 20. Jahrhunderts an und ergab sich zunächst allem voran aus den technischen Bedingungen der zunehmend auf die Bedeutung von Keimdrüsen konzentrierten Experimentalsysteme der frühen embryologischen Geschlechtsentwicklungsforschung. Damit wird eine Forschungslücke aus früheren Studien adressiert – etwa von Marianne van den Wijngaard (1997) und Ulrike Klöppel (2006; 2008) –, die zwar ebenfalls die Bedeutung experimenteller Praktiken für die Entstehung von Wissen über die Geschlechtsentwicklung thematisierten, die frühe tierexperimentelle endokrinologische Forschung vor Ende der 1940er Jahre jedoch noch nicht systematisch in den Blick nahmen. Um diese Lücke zu schließen, werden auf Grundlage von Fachartikeln sowie wissenschaftlichen Monografien vier zentrale Experimentalsysteme aus dem Zeitraum 1900 bis 1950 untersucht, in denen der Prozess der pränatalen Geschlechtsentwicklung erstmals mit den Wirkungen von Hormonen assoziiert wurde und die deshalb als wichtige Vorläufer der modernen endokrinologischen Geschlechtsentwicklungsforschung angesehen werden können: (1) die anatomisch-morphologischen Untersuchungen von embryonalem Keimdrüsengewebe der französischen Anatomen Pol Bouin und Paul Ancel um 1900, (2) die Freemartin-Forschung in Österreich und den USA in den 1910er Jahren, (3) die etwa zeitgleichen Geschlechtsumwandlungsexperimente an Nagetieren des österreichischen Physiologen Eugen Steinach, sowie (4) die Geschlechtsentwicklungsforschung mit Sexualhormonen der heute weitgehend vergessenen, jedoch zu Lebzeiten renommierten russisch-US-amerikanischen Embryologin Vera Dantschakoff in den 1930er Jahren.

Diese Analyse öffnet den Raum für die Diskussion von möglichen Pfadabhängigkeiten, die zwischen den Experimentalsystemen sowie Theorien über Geschlecht der zweiten Hälfte des 20. Jahrhunderts und vorangehenden experimentellen Praktiken und Theorien bestehen. Aber welchen Mehrwert verspricht ein historischer Rekurs auf die materiell-technischen Bedingungen von zurückliegenden Experimentalsystemen, und was bedeutet „Pfadabhängigkeit“ in diesem Kontext? Nach Alexandra Waluszewski und Håkan Håkansson (2002: 561) kann „[e]ine Pfadabhängigkeit […] als etwas verstanden werden, das einschränkt, was getan werden kann.“ Sie erklären, dass „Pfadabhängigkeit nicht nur ein integraler Bestandteil des Wissens, sondern auch […] aller technischen Elemente“ sei, wobei Systeme mit der Zeit die Fähigkeit zu grundlegenden Veränderungen einbüßen würden. Daraus folgern sie: „[D]ieser Prozess der ‚Schließung‘ impliziert, dass die Flexibilität des Systems abnimmt“ (ebd.: 561). Eine solche „Schließung“, so noch einmal die zentrale These des Artikels, ergab sich im Bereich der Geschlechtsentwicklungsforschung bereits im Rahmen der frühesten endokrinologischen Experimentalsysteme, die Alfred Josts experimentellen Arbeiten Ende der 1940er Jahre vorangingen. Die erfolgreiche experimentelle Operationalisierung der männlichen Geschlechtsentwicklung im Rahmen dieser Experimentalsysteme lenkte die Forschung dabei in eine Bahn, die zu verlassen zunehmend schwierig wurde, da sich nicht nur auf argumentativer, sondern auch auf materiell-technischer Ebene eine Androgen-Zentrierung einstellte.

In Anschluss an Hans-Jörg Rheinbergers „historische Epistemologie des Experiments“ stehen die Experimentalsysteme der frühen Geschlechtsentwicklungsforschung als Rahmenbedingungen der Hervorbringung einerseits von „epistemischen Dingen“ und andererseits von „technischen Dingen“ im Fokus der folgenden Analyse (Rheinberger 2019: 27).^4^ Unter „epistemischen Dingen“ versteht Rheinberger (2001: 15) „Dinge, in denen sich Begriffe verkörpern“. Im hier betrachteten Fall ist es binäre Geschlechtlichkeit, die sich zu Beginn des 20. Jahrhunderts in neuen Wissensobjekten – den als „männlich“ und „weiblich“ konzipierten Androgenen und Östrogenen – innerhalb von bestimmten Experimentalsystemen zu verkörpern begann. Diese Hormone verblieben dabei jedoch nicht im Status der „epistemischen Dinge“, sondern transformierten sich spätestens in den 1930er Jahren auch in „technische Dinge“ – eine Unterscheidung, die bei Rheinberger (2001: 26) funktional gedacht ist. Als ausreichend stabilisierte und mit Wissen beladene „epistemische Dinge“, die bestimmte Effekte systematisch zu reproduzieren vermögen, konnten Geschlechtshormone aus ihrem ursprünglichen Kontext (einem Organismus) durch pharmaindustrielle Verfügbarmachung entnommen und als Werkzeuge in neuen Experimentalsystemen eingesetzt werden. Sie wurden dabei zu Bestandteilen der technischen Arrangements, in denen Wissen über die embryonale Geschlechtsentwicklung produziert wurde und zeigten hier zum Teil unerwartete Wirkungen: In Vera Dantschakoffs Reflexionen der Experimentverläufe aus den 1930er Jahren wird deutlich, dass Östrogene sich mit der Zeit als widerspenstige Agentien und schwer operationalisierbare „technische Dinge“ in den Experimentalsystemen der embryologischen Geschlechtsentwicklungsforschung herausstellten. Anders als Androgene, die in Experimenten an Säugetieren systematisch Effekte der Vermännlichung hervorriefen, erwiesen sich Östrogene als tödlich für Säugetierembryonen (Dantschakoff 1938: 149). Dieser materiell-technische Umstand könnte eine entscheidende Rolle für die zunehmende Fokussierung auf den Einsatz von Androgenen in der Geschlechtsentwicklungsforschung sowie die daraus hervorgehende Theoriebildung gespielt haben. Schließlich bilden laut Rheinberger die technischen Bedingungen den produktiven Rahmen für die Entstehung von Wissensobjekten „und lassen sie so erst als solche hervortreten, sie begrenzen sie aber auch und schränken sie ein“ (Rheinberger 2001: 26).

Der vorliegende Beitrag nimmt ebensolche materiell-technischen Bedingungen mitsamt ihrer Begrenzungen in Prozessen der experimentellen Operationalisierung der pränatalen Geschlechtsdifferenzierung in den Blick und ist anachronistisch aufgebaut: Er setzt in den 1940er Jahren an und gibt zunächst einen schlaglichtartigen Überblick der Entwicklungen im Bereich der embryonalen Geschlechtsentwicklungsforschung seit Ende der 1940er Jahre bis in die Gegenwart – und damit der Ereignisse, die seit den 1980er Jahren im Fokus der feministischen Kritik stehen. Darauf folgt der empirische Teil der Studie und damit ein zeitlicher Sprung an den Beginn des 20. Jahrhunderts. Bei der Analyse der vier Experimentalsysteme sind folgende Fragen forschungsleitend: Welche experimentellen Methoden wurden angewandt? Welche Aussagen über Geschlecht und die Geschlechtsentwicklung wurden dabei generiert? Welche Beschränkungen des Wissens ergaben sich aus den materiell-technischen Bedingungen dieser Forschung und welche Konsequenzen kann dies für die weitere Produktion von Wissen im Bereich der pränatalen Geschlechtsentwicklungsforschung gehabt haben? Das Erkunden der produktiven wie restriktiven Dimensionen der endokrinologischen Experimentalsysteme im Zeitraum von 1900 bis 1950 soll aufschlussreiche Einblicke in die sogenannte *black box *der zurückliegenden Forschungsprozesse liefern, womit das zunehmende Unsichtbarwerden der eigentlichen wissenschaftlichen Arbeit und ihrer technischen Bedingungen zugunsten von dabei produzierten Forschungsergebnissen gemeint ist (Latour & Woolgar 1986: 242). Auf diesem Wege soll auch im Hinblick auf aktuelle, politisch aufgeladene Debatten um die wissenschaftliche Produktion von Geschlechterwissen verdeutlicht werden, dass ein feministisch-informiertes Umdenken in den Biowissenschaften nicht nur das Überwinden von tief mit kulturell verankerten Geschlechterstereotypen verwobenen Denkmustern erfordert, sondern auch eine Transformation von historisch gewachsenen und diese Stereotype stetig reproduzierenden materiell-technischen Bedingungen der biomedizinischen Forschung.

## Theorien zur embryonalen Geschlechtsentwicklung seit den 1940er Jahren

Als Begründer des bis heute gültigen Konzepts der „primären Weiblichkeit“ und einer der einflussreichsten Vertreter der Androgen-zentrierten Geschlechtsentwicklungsforschung in der vormolekularen Ära gilt der französische Embryologe Alfred Jost (Zhao & Yao 2019: 602). Seine Forschung adressierte die Frage nach den Mechanismen der embryonalen Geschlechtsdifferenzierung von Säugetieren abseits von genetischen Faktoren und baute auf der bereits in den vorhergehenden vier Jahrzehnten etablierten Annahme, dass die embryonalen Keimdrüsen eine Rolle in diesen Prozessen spielen könnten (Jost 1947a: 151–152). Konkret experimentierte Jost in den 1940er Jahren mit Kaninchenembryonen und wandte eine zu dieser Zeit neuartige Operationstechnik an, die auf die Kastration ihrer Keimdrüsen zielte. Sie erlaubte es, die zunächst geschlechtlich undifferenzierten Keimdrüsenanlagen von Embryonen in der sogenannten „kritischen Phase“ zu entfernen – also bevor sie sich in Ovarien- oder Hodengewebe zu entwickeln begannen.^5^ Aus diesen Experimenten wurden folgende Erkenntnisse gewonnen: Nach der operativen Entfernung des noch undifferenzierten Keimdrüsengewebes entwickelten sich die bipotenten Geschlechtsanlagen aller Embryonen – unabhängig von ihrem chromosomalen Geschlecht – in eine weibliche Richtung, so Jost (1948: 208). Die Applikation einer geringen Menge synthetischen Androgens zum Zeitpunkt der fötalen Kastration sollte wiederum die Differenzierung der Geschlechtsorgananlagen aller Tiere in eine männliche Richtung initiieren (ebd.: 211). Für Jost war dies ein entscheidender Verweis auf die zentrale Bedeutung von Androgenen für die männliche Geschlechtsentwicklung (ebd.: 229). Gleichzeitig schlossen diese Experimentalergebnisse ihm zufolge nicht gänzlich aus, dass die embryonalen Ovarien ebenfalls in irgendeiner Form die weibliche Geschlechtsentwicklung steuerten (ebd.: 224, 229). Dies sollte aus seiner Sicht in Zukunft im Rahmen neuer experimenteller Ansätze überprüft werden. Eine solche Überprüfung rückte jedoch rasch in den Hintergrund einer sich zunehmend dynamisierenden und auf die Wirkungen von Androgenen konzentrierten Geschlechtsentwicklungsforschung, die spätestens mit Josts Experimenten eine solide Grundlage hatte und ebenso rasch neue Ergebnisse produzierte wie sie neue Detailfragen in Zusammenhang mit der männlichen Geschlechtsdifferenzierung aufwarf (Fausto-Sterling 2000: 203, 347). So zeigten Josts Experimente beispielsweise, dass die durch Androgene induzierte männliche Differenzierung der Geschlechtsanlagen von Kaninchenembryonen nicht vollständig der natürlichen Entwicklung entsprach, da die Versuchstiere parallel zu den männlichen auch rudimentäre weibliche Geschlechtsanlagen ausbildeten (Jost 1948: 208). Dies veranlasste ihn zur Hypothese eines weiteren, vom Hoden produzierten und die weibliche Entwicklung hemmenden Faktors, den er als „hormone inhibitrice“ oder „antifeminine factor“ bezeichnete und dessen Aufklärung die embryologische Geschlechtsentwicklungsforschung bis in die 1980er Jahre beschäftigen sollte (Josso 1986: 421).

Während die männliche embryonale Geschlechtsentwicklung in der zweiten Hälfte des 20. Jahrhunderts also zunehmend detailliert als Folge der Wirkungen von spezifisch männlichen hormonellen Faktoren beschrieben wurde und sich ein international expandierendes Forschungsfeld zur Untersuchung dieses Mechanismus und generell der Erforschung von Androgenen bildete,^6^ blieben die Mechanismen der weiblichen embryonalen Geschlechtsentwicklung eine Leerstelle. Die in den Publikationen aus den 1940er Jahren noch deutlich geäußerte Unklarheit darüber, ob die embryonalen Ovarien und ihre Hormone tatsächlich keine Rolle bei der weiblichen Geschlechtsdifferenzierung spielten, geriet dabei zunehmend in den Hintergrund der Forschung – eine Entwicklung, die sich recht anschaulich an den Publikationen von Alfred Jost im Zeitraum zwischen 1950 und 1965 nachvollziehen lässt.^7^ Anstatt nach neuen Methoden zur Untersuchung der weiblichen Geschlechtsdifferenzierung zu suchen, war die Forschung zur pränatalen Geschlechtsentwicklung fundamental durch die hormonelle „An- und Abwesenheitstheorie“ geprägt, die die Leerstelle des Wissens um die weibliche Geschlechtsentwicklung mit einer Ex-negativo-Theorie der Abwesenheit von Androgenen gefüllt hatte. Diese Theorie wurde Ende der 1950er Jahre im Kontext der Verhaltensforschung weiter ausbuchstabiert.

Mit der sogenannten „Organisationshypothese“ eines US-amerikanischen Forschungsteams um die Biologen Charles H. Phoenix und William C. Young aus dem Jahr 1959 wurde das Konzept der An- und Abwesenheit von Androgenen zu einem zentralen Dreh- und Angelpunkt einer zunehmend interdisziplinär aufgestellten tierexperimentellen Geschlechtsentwicklungsforschung (Doell & Longino 1988; van den Wijngaard 1997). Sie resultierte aus einer Verknüpfung von Alfred Josts Postulat der Androgen-abhängigen pränatalen Entwicklung der Geschlechtsorgane mit Beobachtungen im Rahmen der Verhaltensforschung an Nagetieren: „Typisch“ männliches Sexualverhalten sowie eine „normale“ männliche sexuelle Orientierung von ausgewachsenen Organismen sollten sich infolge von pränatalen Androgen-Einwirkungen auf neuronales Gewebe einstellen, das im späteren Leben das Paarungsverhalten vermittelte (Phoenix et al. 1959). Damit wurde die An- oder Abwesenheit von „männlichen“ Geschlechtshormonen während der embryonalen Entwicklung zum entscheidenden Faktor nicht nur für den Aufbau von geschlechtsspezifischen Geschlechtsorganen, sondern auch für eine geschlechtsspezifische Entwicklung von Gehirn- und Nervensystem sowie eines daran gekoppelten geschlechtsspezifischen Paarungsverhaltens erhoben (Wallen 2009: 562–653).

Diese aus Verhaltensexperimenten an Meerschweinchen stammende Organisationshypothese war zunächst durchaus umstritten und wurde vielfach an anderen nicht-menschlichen Spezies, darunter auch Primaten, mit teils widersprüchlichen Ergebnissen überprüft (ebd.: 564). Mit den Arbeiten der beiden Psycholog:innen Anke Ehrhardt und John Money aus den 1970er Jahren wurde sie schließlich auch auf Menschen übertragen und auf Fragestellungen jenseits des Sexualverhaltens ausgeweitet (Money & Ehrhardt 1972). Ehrhardt und Money vertraten die These, dass die An- oder Abwesenheit von pränatalen Androgenen entscheidend für die spätere Entwicklung des gesamten Repertoires von als männlich oder weiblich gelesenen Verhaltensweisen sein musste. Begründet wurde dies mit Verhaltensbeobachtungen an intersexuellen Kindern (Eder 2011; Jordan-Young 2010: 29–36; Klöppel 2008; 2010: 495–499; van den Wijngaard 1997: 31–32). Zeigten genetisch weibliche Kinder mit uneindeutigen Geschlechtsorganen Verhaltensweisen wie „rough and tumble play“, was sich am ehesten mit „wilde, körperbetonte Spiele“ übersetzen lässt und von den Autor:innen als typisch männliches Spielverhalten gedeutet wurde, so vermutete man aus diesen Beobachtungen heraus, dass die Gehirne dieser Kinder parallel zu ihren Genitalien in embryonalen Entwicklungsstadien durch Androgene maskulinisiert worden waren (Money & Ehrhardt 1972).

Im Umkehrschluss, so postulierte es zur gleichen Zeit der ostdeutsche Sexualendokrinologe Günter Dörner, würde männliche Homosexualität aus einem Mangel an Androgen-Einwirkung in „kritischen Phasen“ der embryonalen Entwicklung des Gehirns genetisch männlicher Föten resultieren (Dörner 1969). Homosexuelles Verhalten war in dieser Logik die Folge einer Feminisierung männlicher Gehirne im Sinne eines pathologischen Verbleibens in einem nicht durch Keimdrüsenhormone differenzierten – ergo weiblichen – Zustand. Ausgehend von dieser im Tierexperiment gewonnenen These, so Florian Mildenberger, propagierte Dörner präventive Androgen-Behandlungen „von Schwangeren zur Heterosexualisierung der sozialistischen Gesellschaft“ (Mildenberger 2006: 258). Trotz der wachsenden Kritik an Dörners Biologisierung und Pathologisierung von Homosexualität trug dieser mit seiner international vielbeachteten tierexperimentellen Sexualhormonforschung und vor allem seinen Übertragungen der an Ratten gewonnenen Forschungsergebnisse auf Menschen maßgeblich zur weiteren Verfestigung der Androgen-zentrierten Theorie der geschlechtsspezifischen embryonalen Differenzierung bei (Bernabé & Giri 2021).

Die Fokussierung auf Faktoren der männlichen Geschlechtsdifferenzierung setzte sich auch im Rahmen der seit den 1980er Jahren zunehmend molekularbiologisch ausgerichteten Genetik der Geschlechtsentwicklungsforschung fort (Richardson 2015: 125–136). Diese widmete sich den bis dato noch unerforschten Mechanismen der primären Differenzierung der zunächst bipotenten Keimdrüsenanlagen in embryonales Ovarien- oder Hodengewebe. Auch dieser Mechanismus wurde kausaldeterministisch als aktive Regulation der Zelldifferenzierung von einem spezifisch männlichen Transkriptionsfaktor – dem sogenannten „Testis Determinierenden Faktor“ – beschrieben, dessen Produktion von der Anwesenheit des als „männlich“ markierten Geschlechtschromosoms Y abhängig sein sollte (Page et al. 1987). Der Molekularbiologe David Page hatte „[d]ie An- oder Abwesenheit des Y‑Chromosoms“ als zentralen und einzigen Faktor beschrieben, der angeblich bestimmte, „ob sich ein Säugetierembryo zu einem männlichen oder weiblichen Tier entwickelt“ (Page et al. 1987: 1091). Die primäre weibliche Geschlechtsdifferenzierung wurde dabei also genau wie die sekundäre als Vorgang konzipiert, der ohne geschlechtsspezifische Faktoren und quasi automatisch ablaufen sollte.

Die genannten Androgen- oder Geschlechtschromosomen-zentrierten Modelle der embryonalen Geschlechtsdifferenzierung des 20. Jahrhunderts werden seit den 2000er Jahren zunehmend von komplexen multifaktoriellen Wirkungsmodellen abgelöst (Berenbaum 2018; Neumann-Held & Rehmann-Sutter 2006; Stévant et al. 2018). Ebenso stellen neuere molekularbiologische Untersuchungen die These der Ovarien-Entwicklung als voreingestellten unspezifischen Mechanismus infrage (Yang et al. 2010; Yao 2005).^8^ Nicht zuletzt häuft sich die feministisch informierte Kritik am Androgen-Zentrismus der Geschlechtsentwicklungsforschung, die auch konkrete Gegenentwürfe vorschlägt (Holterhus & Hiort 2021: 383; Graves 2000; Schiebinger et al. 2011–2020). Dennoch zeigt sich eine enorme Beharrungstendenz in der Konzeptualisierung der weiblichen Geschlechtsdifferenzierung als unspezifischem Entwicklungsprozess. So heißt es beispielsweise in der letzten Auflage eines im Jahr 2025 als zentrales Lehrwerk geltenden Lehrbuchs der Embryologie für medizinische Studienfächer im deutschsprachigen Raum auch weiterhin:

Die primäre weibliche Differenzierung hängt möglichweise garnicht von besonderen weiblichen Faktoren oder Hormonen ab; sie findet zum großen Teil sogar statt, sobald das Y‑Chromosom fehlt, sodass man bei der weiblichen Entwicklung vom „default type“ (also dem „voreingestellten“ Typ) spricht. (Persaud & Torchia 2026: 298)

Die folgenden Abschnitte blicken auf die Vorgeschichte der bisher geschilderten Ereignisse und somit auf die Anfänge der embryonalen Geschlechtsentwicklungsforschung in den ersten drei Dekaden des 20. Jahrhunderts und speziell die materiell-technischen Bedingungen dieser Forschung.

## Keimdrüsen und Geschlecht: Zu den Anfängen der embryonalen Geschlechtsentwicklungsforschung um 1900

„Wir wissen, dass das Genitale im Embryo hermaphroditisch angelegt ist“, konstatierte der österreichische Gynäkologe Josef Halban im Jahr 1903 und führte weiter aus:

Da nun die Differenzierung der Keimdrüse der Differenzierung der Wolff’schen und Müller’schen Organe weit vorauseilt, und da man weiter gefunden hat, dass dann, wenn ein Hode entsteht, die männlichen Antheile [sic!] des Genitales sich entwickeln und die weiblichen zurückbleiben, wenn aber ein Ovarium entsteht, die weiblichen sich entwickeln und die männlichen zurückbleiben, so lag die Vermutung nahe, dass die Keimdrüse einen morphogenetischen oder formativen Reiz auf die Entwicklung der entsprechenden Genitalantheile [sic!] ausübt. (Halban 1903: 206)

Der Begriff „hermaphroditisch“ stand dabei für die Annahme, dass Embryonen zunächst sowohl männliche als auch weibliche geschlechtliche Anlagen nebeneinander enthielten, von denen jeweils nur eine zur vollen Ausprägung gelangte (Klöppel 2010: 247). Anders als bei Halban herausklingt, war diese These aber durchaus umstritten und wurde schon bald von der Vorstellung einer gemeinsamen „indifferenten geschlechtlichen Anlage“ verdrängt (Voß 2010: 156).^9^ Ungeachtet der Uneinigkeit über den embryonalen Urzustand der Geschlechtsanlagen herrschte um 1900 bereits weitgehender Konsens darüber, dass es die embryonalen männlichen und weiblichen Keimdrüsen sein mussten, die über „formative Wirkungen“ (Halban 1903: 211) zum weiteren Aufbau des Geschlechts verfügten. Dieser Konsens speiste sich aus bis in die Antike zurückreichenden Urmotiven der Hoden als Sitz von Männlichkeit sowie einer diskursiven Verknüpfung der Gebärmutter mit Weiblichkeit, die im 19. Jahrhundert durch das Postulat der Weiblichkeit als „eine Dependenz des Eierstocks“ abgelöst wurde (Virchow 1856: 747).^10^ Die in der zweiten Hälfte des 19. Jahrhunderts allmählich aufgekommene neue physiologische Lehre von der „Inneren Sekretion“ – also die Vorstellung von über den Körper verteilten Drüsen, die Stoffe in das Körperinnere abgeben, welche wiederum an anderen Orten des Organismus regulatorische Wirkungen entfalten – fügte sich nahtlos in diese kulturell längst verankerten Motive ein und erweiterte sie um eine physiologisch-theoretische sowie experimentell greifbare materielle Ebene.^11^

Im Fall der Keimdrüsen richtete sich der forschende Blick der Physiologie neben der experimentellen Untersuchung ihrer Wirkungen auch auf den konkreten Entstehungsort der wirksamen Substanzen – seit 1905 bezeichnet als „Hormone“ – innerhalb des Keimdrüsengewebes. Möglich wurde dies durch histologische Färbungen und anschließende morphologische Untersuchungen von Gewebeproben unter dem Mikroskop. Die männlichen Keimdrüsen standen dabei in besonderem Fokus, da es einen wissenschaftlichen Streit darüber gab, ob die spermatogenen Elemente nur für die Reproduktion oder auch für die Ausbildung sämtlicher geschlechtsspezifischer Merkmale verantwortlich waren – also die Funktion der „äußeren“ und „inneren Sekretion“ in sich vereinten (Stoff 2004: 37). Die histologischen Untersuchungen der beiden Franzosen Pol Bouin und Paul Ancel um die Jahrhundertwende zum 20. Jahrhundert führten nicht nur zu einer differenzierteren Unterteilung von Hodengewebe in eine Sperma-produzierende Drüse mit äußerer und eine sogenannte „Interstitielle Drüse“ mit innerer Sekretion (Bouin & Ancel 1903a). Ihre gemeinsamen Arbeiten markieren auch den historischen Moment der Dynamisierung einer explizit endokrinologischen Embryologie, die sich in den darauffolgenden Jahrzenten auf die frühen geschlechtsdifferenzierenden Wirkungen der als „männlich“ und „weiblich“ konzipierten Keimdrüsenhormone – der Androgene und Östrogene – konzentrieren sollte. Bezeichnend für diesen Moment und für unsere Fragestellung ist, dass sich bereits in diesen frühesten Untersuchungen eine Widerspenstigkeit der weiblichen embryonalen Geschlechtsdifferenzierung gegenüber den endokrinologischen Experimentalsystemen abzeichnet.

## Experimentalsystem I: Keimdrüsenhormone und männliche Geschlechtsentwicklung – Die Forschung von Pol Bouin und Paul Ancel um 1900

Im Jahr 1903 veröffentlichten die Anatomen Pol Bouin und Paul Ancel mehrere wegweisende Publikationen, die bis heute in der historischen Rückschau auf die Anfänge der endokrinologischen Geschlechtsentwicklungsforschung als zentrale Referenzen gelten (Smith & McEwan 2013). Eine dieser Studien basierte auf histologisch-morphologischen Untersuchungen von Ovarien und Hoden weiblicher und männlicher Schweineembryonen (Bouin & Ancel 1903b). In einem histologischen Laboratorium der medizinischen Fakultät im französischen Nancy nutzten die beiden Anatomen ein Experimentalsystem, das auf seit Mitte des 19. Jahrhunderts entwickelten und stetig verfeinerten Techniken der Anfärbung von histologischen Gewebeschnitten beruhte (Lang 2006: 415).^12^ Im Gegensatz zu späteren – im eigentlichen Sinne *experimentellen* – Ansätzen der Endokrinologie griffen sie nicht aktiv in Entwicklungsprozesse ein, sondern analysierten unbeeinflusstes embryonales Hoden- und Ovariengewebe in verschiedenen Entwicklungsstadien, das mit selektiven Farbstoffen präpariert und anschließend mikroskopisch untersucht wurde.

Obwohl die einzelnen Gewebeschnitte lediglich Momentaufnahmen physiologischer Prozesse darstellten, ermöglichte ihre Entnahme in unterschiedlichen embryonalen Entwicklungsstadien und der Vergleich des morphologischen Zustands dieser Präparate Rückschlüsse auf die ablaufenden zellulären Entwicklungen. Die Befunde wurden in Referenz zu zeitgenössischen physiologischen Theorien gedeutet – insbesondere zu der von Bouin und Ancel (1903a) selbst postulierten Annahme einer innersekretorischen Funktion interstitieller Zellen im Hodengewebe. Sie beobachteten, dass diese Zellen im frühen embryonalen Stadium in der mikroskopischen Betrachtung besonders voluminös erschienen – und dies just zu jenem Zeitpunkt, an dem die erste Differenzierung männlicher Geschlechtsorgane mikroskopisch sichtbar wurde. Daraus folgerten die beiden Anatomen, dass ein bislang unbekannter, von diesen Zellen sezernierter „männlicher“ Keimdrüsenwirkstoff für die Initiierung der männlichen Geschlechtsentwicklung verantwortlich sein musste (Bouin & Ancel 1903b: 1684). Die zu dieser Zeit noch nicht näher beschriebenen „männlichen“ Keimdrüsenwirkstoffe – „epistemische Dinge“ und zentrale Wissensobjekte dieser Forschung – wurden als entscheidende Agentien der Geschlechtsentwicklung konzipiert, deren *Anwesenheit* und Wirkung entscheidend für die Ausbildung männlicher Geschlechtsmerkmale sein sollte.

Welche Aussagen aber brachte dieses Experimentalsystem über die weibliche embryonale Geschlechtsentwicklung hervor? Dieser Punkt ist von besonderem Interesse, denn die beiden Anatomen äußerten sich dazu folgendermaßen:

Tatsächlich gibt es keine interstitiellen Zellen während der ersten ontogenetischen Phasen des Eierstocks; man findet nichts zwischen den jungen Pflügerschnüren und unterhalb des Keimepithels, das an die interstitielle Drüse erinnert, wie sie sich während der entsprechenden Phasen der Hodendifferenzierung entwickelt. (Bouin & Ancel 1903b: 1682)

Die „augenscheinliche“ *Abwesenheit* von weiblichem Keimdrüsengewebe in frühen embryonalen Differenzierungsstadien wurde von Bouin und Ancel nicht weiter interpretiert, sodass die weibliche Geschlechtsentwicklung und mögliche „weibliche“ Keimdrüsenwirkstoffe hier epistemisch und dinglich unbestimmt blieben. Die materiell-technischen Bedingungen dieses sehr frühen – im weitesten Sinne endokrinologischen – Experimentalsystems stellten mit Rheinberger (2001: 26) also zwar einen produktiven Rahmen für die Formierung von Wissensobjekten in Zusammenhang mit der männlichen Geschlechtsentwicklung dar. Sie schränkten jedoch die Produktion von Wissen über die weibliche Geschlechtsentwicklung ein, die in ihrem Rahmen ungreifbar blieb.

Zusammenfassend lässt sich sagen, dass die anatomisch-morphologischen Forschungen von Bouin und Ancel eine theoretische Basis für Experimentalsysteme lieferten, die sich in Folge mit der Erforschung der embryonalen Geschlechtsdifferenzierung explizit in Zusammenhang mit den zunächst noch gänzlich hypothetischen Wirkstoffen der Keimdrüsen widmeten. Sie markieren einen frühen Punkt auf dem Pfad hin zu den zunehmend elaborierten und als „aktive Wissenschaft“ agierenden endokrinologischen Experimentalsystemen der zweiten Hälfte des 20. Jahrhunderts und liefern zugleich einen ersten Spin in Richtung Androgen-Zentrismus. Dieser speiste sich hier allerdings noch nicht aus einer spezifischen Interpretation, sondern vielmehr aus dem materiell-technischen Umstand der Unsichtbarkeit – ergo optischen *Abwesenheit* – von Agentien, die den Prozess der pränatalen Differenzierung von weiblichen Geschlechtsorganen vermitteln könnten.

## Experimentalsystem II: Ein intersexueller Modellorganismus – Die Freemartin-Forschung der 1910er Jahre

Einen weiteren Schritt in Richtung Androgen-Zentrismus in der embryologischen Geschlechtsentwicklungsforschung stellen Studien aus den 1910er Jahren dar, deren Erkenntnisgewinn ebenfalls auf anatomisch-morphologischen Untersuchungen fußte und in deren Fokus ungewöhnliche Modellorganismen standen: die sogenannten „Freemartins“, zu Deutsch „Zwicken“. Der Freemartinismus ist ein Phänomen, das bereits im 17. Jahrhundert beschrieben und im Rahmen der Nutztierhaltung meist bei Rindern beobachtet wurde (Capel & Coveney 2004: 853). Dabei handelt es sich um genetisch weibliche Tiere aus Zwillings- oder Mehrlingsgeburten, die mit Fehlbildungen der Geschlechtsorgane geboren werden und steril sind. Die Geschlechtsorgane solcher Tiere sind als Mischformen aus äußeren weiblichen und inneren männlichen Organen ausgebildet, weshalb Freemartins zu Beginn des 20. Jahrhunderts als „hermaphroditisch“ oder auch „intersexuell“ bezeichnet wurden (Goldschmidt 1917: 442). Wie es in vielen Publikationen dazu heißt, sei diese Intersexualität bei Rindern durch ein „Naturexperiment“^13^ (ebd.: 442) hervorgebracht worden und erregte in den ersten Dekaden des 20. Jahrhunderts großes Forschungsinteresse – zum einen seitens der veterinärmedizinischen Forschung, die landwirtschaftliche Interessen verfolgte, zum anderen seitens der Embryologie, die ausgehend von dieser systematisch auftretenden Geschlechtsanomalie etwas über die Mechanismen lernen wollte, die die Geschlechtsdifferenzierung im Embryonalstadium vermittelten (Fausto-Sterling 2000: 163–177).

So waren es zwei Forschungsgruppen aus Österreich und den USA, die in den 1910er Jahren unabhängig voneinander zur selben Hypothese über die Ursache des Freemartinismus gelangten: Sie postulierten, dass „männliche“ Geschlechtshormone für die Maskulinisierung genetisch weiblicher Föten verantwortlich seien (Freeman 2007). Beide Gruppen nutzten in ihren Experimentalsystemen anatomisch-morphologische Methoden, näherten sich dem Phänomen des Freemartinismus jedoch ausgehend von unterschiedlichen Fragestellungen an. Der Veterinärmediziner Karl Keller und sein Kollege, der Mediziner und Anatom Julius Tandler, forschten am 1. Anatomischen Institut der Universität Wien und interessierten sich für das Phänomen der natürlichen Kastration in Embryonen (ebd.: 106). In einer vorläufigen Mitteilung auf Grundlage anatomischer Untersuchungen der Eihäute bei Zwillingsgraviditäten des Rindes formulierten sie bereits im Jahr 1911 die Hypothese, dass vaskuläre Anastomosen zwischen den Plazenten heterosexueller Zwillinge eine hormonelle oder zelluläre Beeinflussung der weiblichen Frucht bewirken könnten (Keller & Tandler 1911). Wahrscheinlich ohne Kenntnis von dieser Publikation zu haben (Freeman 2007), bestätigte und erweiterte der US-amerikanische Zoologe Frank R. Lillie diese Annahme durch eine systematische Reihe histologischer Analysen an einem umfangreichen Korpus von Zwillingspaaren, die er in Zusammenarbeit mit Viehzüchter:innen aus geschlachteten trächtigen Kühen gewinnen ließ (Lillie 1917: 378–379). Im Gegensatz zu Tandler und Keller galt sein Interesse nicht den Ursachen der Unfruchtbarkeit von „Zwicken“, sondern vielmehr den physiologischen Mechanismen der Umsetzung einer genetisch festgelegten geschlechtlichen Veranlagung (Salomon 2025). Er schreibt:

Wenn beide [Zwillinge] männlich oder beide weiblich sind, hat dies keine negativen Auswirkungen; *wenn jedoch einer männlich und der andere weiblich ist, werden die Reproduktionsorgane des Weibchens weitgehend unterdrückt und es entwickeln sich sogar spezifisch männliche Organe im Weibchen. Dies ist zweifellos als ein Fall von Hormonwirkung zu interpretieren *[H. i. O.] (Lillie 1916: 612).

Keimdrüsenhormone von männlichen Embryonen würden auf dem Weg über sogenannte Anastomosen – also über die Plazenta verbundene gemeinsame Blutgefäße der gemischtgeschlechtlichen Embryonen – in den Organismus der weiblichen Tiere gelangen und dort Störungen der Geschlechtsentwicklung verursachen, so die Hypothese (Lillie 1916: 612). Diese Schlussfolgerung begründeten sowohl Lillie als auch Tandler und Keller mit der Beobachtung, dass der vermännlichende Effekt nur dann auftrat, wenn sich embryonale Anastomosen zwischen den Zwillingen bildeten – ergo über den Blutkreislauf zirkulierende geschlechtsspezifische Wirkstoffe am Werk sein mussten (Keller & Tandler 1916: 526). Ob der maskulinisierende Effekt sich aus einem früheren Einsetzen der männlichen Keimdrüsenhormonproduktion ergab oder aus einer grundsätzlichen „natürlichen Dominanz von männlichen über weibliche Hormone“ sei noch unklar, so Lillie (1916: 612). Ebenso blieb weiterhin offen, um welche hormonellen Substanzen es sich bei den Wirkstoffen der embryonalen männlichen Keimdrüsen genau handeln könnte.^14^

Trotz der auch weiterhin nur schemenhaften Vorstellungen von den Mechanismen der embryonalen Geschlechtsdifferenzierung erhärteten diese Untersuchungen die von Bouin und Ancel postulierte Theorie einer hormonellen Regulation der pränatalen Geschlechtsentwicklung. Das Experimentalsystem der Freemartin-Forschung mit seinen spezifischen materiell-technischen Bedingungen produzierte zudem erneut ausschließlich Aussagen über die männliche Geschlechtsentwicklung und stabilisierte lediglich „männliche“ Hormone als „epistemische Dinge“ der Geschlechtsentwicklungsforschung, da der zentrale Modellorganismus einen Fall der Vermännlichung darstellte. Ihm stand kein entsprechender Modellorganismus gegenüber, der Verweiblichung repräsentiert hätte und weibliche Hormone oder andere Faktoren der weiblichen Geschlechtsdifferenzierung als „epistemische Dinge“ adressierte. So trugen diese Forschungen zwar zur „Chemikalisierung des Geschlechts“ (Oudshoorn 1995: 153) bei, indem sie Geschlecht hormonell fassten, die weibliche Bedingung klammerten sie jedoch erneut aus.

Mit den Geschlechtsumwandlungsexperimenten des Zoologen Eugen Steinach avancierten schließlich auch weibliche Geschlechtshormone zu „epistemischen Dingen“ der Geschlechtsentwicklungsforschung. Steinachs Untersuchungen basierten dabei auf experimentellen Praktiken, die nunmehr im Modus der „aktiven Wissenschaft“ operierten, da er seine Modellorganismen einer systematischen experimentellen Manipulation und willkürlichen Transformation ihrer Geschlechtsmerkmale unterzog.

## Experimentalsystem III: Verweiblichte Männchen und vermännlichte Weibchen – Die Experimente von Eugen Steinach in den 1910er Jahren

Die 1910er Jahre bereiteten dies- und jenseits des Atlantiks eine bald beginnende „goldene Ära“ der Endokrinologie vor (Fausto-Sterling 2000: 170) – eine Zeit, in der die Hormonlehre nicht nur große Hoffnungen als umfassendes neues Erklärungsmodell für zahlreiche Erkrankungen weckte, sondern auch zur Projektionsfläche für teils fantastische Machbarkeitsvorstellungen wurde (Steinbach 2023: 13). Eine wichtige Rolle spielten dafür die international vielbeachteten Geschlechtsumwandlungsexperimente des österreichischen Anatomen und Zoologen Eugen Steinach, in deren Rahmen auch die „weiblichen“ Geschlechtshormone – Östrogene – zu „epistemischen Dingen“ und Objekten der Materialisierung von Geschlecht wurden (Sengoopta 2006; Stoff 2004; Walch 2016). Steinachs Experimentalsystem unterschied sich in zwei Punkten grundlegend von den bisher dargestellten Experimentalsystemen der Geschlechtsentwicklungsforschung. Der erste Punkt betrifft den Modus des Erkenntnisgewinns: Mit François Jacob kann Steinachs Vorgehen als „aktive Wissenschaft“ bezeichnet werden, da er systematisch – oder, wie damals häufig geschrieben wurde, „willkürlich“ (Steinach 1912) – in physiologische Prozesse eingegriffen hat, um „Missgestaltungen“ zu produzieren, aus denen er wiederum Schlüsse auf „normale“ Funktionen zog.^15^ Darüber hinaus war Steinachs Experimentalsystem nicht embryologisch, sondern basierte auf der Entnahme und Verpflanzung von Keimdrüsen bereits geborener Modellorganismen – meist Ratten und Meerschweinchen. Insofern generierten seine Experimente kein Wissen über die pränatale Geschlechtsdifferenzierung, lieferten der späteren embryologischen Geschlechtsentwicklungsforschung jedoch sowohl die materiell-technische Basis für neue und ebenfalls manipulativ operierende Experimente als auch eine epistemische Grundlage für den Einsatz sowohl von „männlichen“ als auch von „weiblichen“ Geschlechtshormonen.

Den theoretischen Ausgangspunkt von Steinachs Experimenten bildete sein Konzept der „Pubertätsdrüse“ – jenem hormonproduzierenden Teil des Hodengewebes, der im Rahmen der histologischen Untersuchungen von Bouin und Ancel bereits als interstitielle Drüse beschrieben worden war (ebd.: 75). Laut Steinachs physiologischer Theorie der postnatalen geschlechtlichen Entwicklung sollten hormonbildende Zellen – ähnlich den interstitiellen Zellen des Hodens – in späteren Entwicklungsstadien auch in den Ovarien weiblicher Säugetiere gebildet werden, um die Entwicklung der sekundären physischen wie psychischen Geschlechtsmerkmale während der geschlechtlichen Reifung zu vermitteln und diese Merkmale anschließend zu erhalten.

Im Jahr 1912 berichtete Steinach von einem Experiment, in dem er etwa drei Wochen alte männliche Meerschweinchen und Ratten kastriert und ihnen anschließend Ovarien eingepflanzt hatte (ebd.). Als Reaktion darauf sollen die Versuchstiere nur rudimentäre männliche Geschlechtsorgane entwickelt und parallel dazu Brustwarzen und Brustdrüsen „in der Form wie bei normalen Weibchen“ aufgewiesen haben (ebd.: 89). Weitere Veränderungen in Richtung Weiblichkeit vermerkte Steinach im Hinblick auf die Behaarung und Körpergröße seiner experimentell „feminierten Männchen“ sowie im Bereich des Psychischen (ebd.: 92–102). Er berichtete von einer „Umstimmung der sexuellen Disposition“ der Versuchstiere, da die „feminierten Männchen“ einen starken Geschlechtstrieb gegenüber anderen Männchen gezeigt haben sollen (ebd.: 103–104). Solche Experimente führte Steinach auch umgekehrt durch, wobei er durch die operative Herstellung von Tieren mit Hoden und gleichzeitig auch Ovarien sogenannte „künstliche Zwitter“ erschuf (Steinach 1913, 1916). War die vorangehende Forschung also noch auf die spontane Entstehung von Intersexualität als „Naturexperiment“ angewiesen, was in Zusammenhang mit Menschen aus ethischen Gründen auch weiterhin der Fall bleiben sollte, so etablierte Steinach die experimentelle Operationalisierung von Intersexualität im Tierversuch. Geschlecht, so zeigte der österreichische Zoologe in seinen Experimenten, war etwas Wandelbares und offenbarte sich in nahezu unzähligen Übergangsformen, die sich in einem Kontinuum zwischen einer idealtypischen Weiblichkeit und Männlichkeit bewegten (Stoff 2021: 197–199). Im Hinblick auf die Experimentalsysteme manifestierte sich mit seinen Transplantationsexperimenten zudem die Vorstellung, Geschlecht sei eine Eigenschaft, die sich aus den Wirkungen von bei Steinach binär und antagonistisch konzipierten geschlechtsspezifischen Keimdrüsenhormonen^16^ ergab und sich demnach mittels operativer Techniken aus einem Organismus herauslösen und in einen anderen übertragen ließ.

Während also die frühe embryologische Geschlechtsentwicklungsforschung bereits seit den anatomisch-morphologischen Studien von Bouin und Ancel und ebenso in der Freemartin-Forschung der 1910er Jahre Tendenzen einer Fokussierung auf die männliche Geschlechtsdifferenzierung zeigte und keine Anhaltspunkte für die Bedeutung „weiblicher“ Keimdrüsenhormone in diesen Prozessen bei Säugetieren gefunden hatte, bestärkten die Experimente Steinachs, dass „weibliche“ Keimdrüsenhormone zumindest in postnatalen Organismen durchaus geschlechtsspezifische Wirkungen zeigen konnten, weshalb auch ihre Relevanz für die embryonale Entwicklung weiterhin nahelag.

Zudem bahnte sich im Rahmen von Steinachs Experimentalsystem bereits die Transformation „männlicher“ und „weiblicher“ Geschlechtshormone von „epistemischen Dingen“ hin zu „technischen Dingen“ an, die später als materiell-technische Bestandteile von Experimentalsystemen der embryologischen Geschlechtsentwicklungsforschung operationalisiert werden konnten. Als in den 1930er Jahren schließlich die Isolierung, Strukturaufklärung und Synthese von Östrogenen und wenig später auch Androgenen erfolgte (Oudshoorn 1994: 76), hatte dies auch weitreichende Auswirkungen auf die endokrinologischen Experimentalsysteme der Geschlechtsentwicklungsforschung und ihre Wissensproduktion. Aufwendige Transplantationsoperationen wurden durch neue Applikationstechniken von pharmaindustriell hergestellten standardisierten Hormonpräparaten ersetzt und die schnellere und kostengünstigere Beschaffung dieser Substanzen eröffnete neue experimentelle Möglichkeiten. Ende der 1930er Jahre formierte sich eine internationale Forschungsgemeinschaft rund um das Thema der embryonalen Geschlechtsdifferenzierung^17^, deren heute nahezu vergessene Schlüsselfigur Vera Dantschakoff^18^ und speziell ihre Arbeiten mit Östrogenen an Säugetierembryonen im Folgenden näher betrachtet werden sollen. In ihnen artikuliert sich ein materiell-technischer Aspekt der Experimentalsysteme zur Erforschung der pränatalen Geschlechtsentwicklung, der historisch bislang keine Beachtung bekam und zugleich eine entscheidende Rolle beim Verschwinden der weiblichen Geschlechtsdifferenzierung von der Agenda der endokrinologischen Forschung im Sinne einer Pfadabhängigkeit der Experimentalsysteme gespielt haben könnte.

## Experimentalsystem IV: Östrogene als widerspenstige Dinge – Die Experimente von Vera Dantschakoff

Zu Beginn der 1930er Jahre standen die Ergebnisse aus den drei skizzierten Experimentalsystemen zunächst nebeneinander: Die histologischen Untersuchungen an Schweineembryonen von Pol Boin und Paul Ancel kurz nach der Jahrhundertwende hatten nahegelegt, dass männliche – jedoch nicht weibliche – Embryonen in einer sehr frühen Phase ihrer Entwicklung erstes geschlechtsspezifisches Drüsengewebe innerhalb ihrer Geschlechtsorgananlagen ausbildeten, welches noch nicht näher bestimmte Drüsenstoffe produzierte. Die Produktion dieser Substanzen sollte zeitlich mit dem Beginn der weiteren Geschlechtsdifferenzierung zusammentreffen, weshalb sie als mögliche histogenetische Werkzeuge dieser Differenzierung betrachtet wurden. Die anatomisch-morphologische Erforschung des Phänomens des Freemartinismus an Föten aus mehrlingsträchtigen Rindern wiederum lieferte Hinweise darauf, dass ebenfalls nicht näher bestimmte Wirkstoffe in der Lage waren, über gemeinsame Blutgefäße zwischen verschiedengeschlechtlichen Zwillingsföten von chromosomal männlichen Organismen in die Körper ihrer genetisch weiblichen Geschwister zu gelangen und sich dort im Sinne einer partiellen „Vermännlichung“ der Geschlechtsorgane auszuwirken – jedoch nicht vice versa. Zeitgleich hatte der Zoologe Eugen Steinach in seinen Geschlechtsumwandlungsexperimenten demonstriert, dass die Verpflanzung von Geschlechtsdrüsen in gegengeschlechtliche Körper bereits geborener Tiere sowohl Effekte einer Vermännlichung als auch einer Verweiblichung von physischen und psychischen Geschlechtsmerkmalen auszulösen vermochte. Die Wirkstoffe der Keimdrüsen ausgewachsener Tiere waren bereits Ende der 1920er Jahre in zunehmend standardisierter Form verfügbar und wurden in den 1930er Jahren auch synthetisch hergestellt. Östrogene und Androgene – nun zunehmend als Sexualhormone bezeichnet – wurden im Rahmen der genannten Experimentalsysteme nicht nur zu weitgehend stabilisierten „epistemischen Dingen“, in denen sich binäre Geschlechtlichkeit zu materialisieren schien. In den 1930er Jahren wurden sie auch zu „technischen Dingen“ der experimentellen Endokrinologie. So wurde schließlich auch das Experimentalsystem der Steinach’schen Geschlechtsumwandlung in der embryologischen Geschlechtsentwicklungsforschung aufgegriffen und auf pränatale Modellorganismen angewandt, was eine Verfeinerung der experimentellen Techniken erforderte.

Es war die russisch-US-amerikanische Embryologin Vera Dantschakoff, die Mitte der 1930er Jahre eine operative Methode der direkten Applikation von Androgenen und Östrogenen in Säugetierembryonen entwickelte, die anschließend auf natürlichem Wege zur Welt gebracht werden konnten (Dantschakoff 1941: 45–47). Nach einem Medizinstudium in Lausanne, einer wissenschaftlichen Promotion und einigen Jahren der histologischen Forschung in Russland migrierte sie im Jahr 1914 in die USA (Rusakova et al. 2020: 9–10; Zubritskiy et al. 2024: 2–3). Im Jahr 1926 kehrte sie in die Sowjetunion zurück und wurde dort zunächst mit einem eigenen Labor betraut, verlor jedoch infolge politischer Repressionen 1932 ihre Forschungsbasis und begann daraufhin eine langjährige Phase nomadischer wissenschaftlicher Tätigkeit an wechselnden Instituten in Kaunas, Bratislava, Berlin, Neapel, Paris und Lausanne, die bis an ihr Lebensende anhalten sollte (Fando 2020). Trotz dieser instabilen Forschungsbedingungen leistete sie maßgebliche Beiträge zur experimentellen Embryologie und etablierte ein innovatives tierexperimentelles System, das sie bis zu ihrem Tod im Lausanne im Jahr 1950 kontinuierlich weiterentwickelte (Rossiianov 2025: 117).

Dantschakoff, die nach eigenen Angaben ein Vivarium bestehend aus einer Vogel‑, Säugetier- Eidechsen‑, Fisch- und Froschabteilung besaß, führte den Großteil ihrer Experimente an weißen und braunen Leghorn-Hühnern und -Hähnen sowie an Meerschweinchen durch (Dantschakoff 1941: 11–51). Auch wenn die bis dato vorliegenden histologisch-anatomischen Untersuchungen an Säugetierembryonen, wie bereits dargelegt, keine Hinweise auf die Bedeutung der weiblichen Keimdrüsenhormone in der embryonalen Geschlechtsentwicklung geliefert hatten und klare geschlechtsdifferenzierende Östrogenwirkungen nur in postnatalen Organismen beschrieben wurden, konnte eine pränatale Rolle der Ovarien und ihrer Hormone auch nicht ausgeschlossen werden. In Dantschakoffs Experimenten mit Vogelembryonen, deren Geschlechtschromosomensätze im Vergleich zu denen von Säugetieren zwischen männlichen und weiblichen Organismen genau umgekehrt verteilt waren, zeigten sowohl Androgene als auch Östrogene geschlechtsdifferenzierende Wirkungen (ebd.: 19–21). Deshalb war auch Dantschakoffs Experimentalsystem mit Säugetieren so konzipiert, dass es die Überprüfung der möglichen Wirkungen beider Geschlechtshormone zuließ. In ihrer umfangreichsten Monografie mit dem Titel *Der Aufbau des Geschlechts beim höheren Wirbeltier* aus dem Jahr 1941 schrieb die zu dieser Zeit bereits Mitte 60-jährige Embryologin, die Behandlung genetisch weiblicher Säugetierembryonen mit Androgenen habe eine deutlich maskulinisierende Wirkung gezeigt, was sie unter anderem an folgenden Kriterien festmachte:

Ein krasserer Gegensatz im äußeren Erscheinen und Verhalten zwischen einem normalen und einem systematisch testosterinierten [mit dem Androgen Testosteron behandelten] Weibchen könnte kaum bestehen. […] Die geschlechtlichen Instinkte sind bei ihnen vollentwickelt, aber in männlicher Richtung: Sie knurren und klappern wie Männchen; ihre unaufhörlichen Kopulationsversuche erweisen sich gelegentlich erfolgreich, indem sie eine Menge von Sekret aus Prostata, Samenblasen, und gelegentlich Uterus ausscheiden. (Dantschakoff 1941: 29)

„‚Arme Fräulein‘“, zitierte Dantschakoff ihre Laborgehilfin Prokofieva, die sich über das aus ihrer Sicht geschlechtswidrige Benehmen der im Embryonalstadium mit Testosteron behandelten Weibchen mokierte: „Daß so eine winzige Menge eingeführten Stoffes ein derartiges perverses Verhalten in ihnen bedingen könnte!“ – ein Kommentar, in dem sich sowohl Ironie als auch die Dominanz heteronormativer Deutungsmuster in dieser Zeit artikuliert (ebd.: 29).

In Zusammenhang mit der Applikation von Östrogenen bei Meerschweinchenembryonen – konkret des damals pharmaindustriell hergestellten Präparats Follikulin – beschrieb Dantschakoff dagegen einen für sie gänzlich unerwarteten Effekt:

[… A]ls ich meine Arbeit an Säugetieren mit Follikulinimpfungen anfing, habe ich viele Enttäuschungen erlebt, da mir nur allmählich klar wurde, daß dieser Stoff vom Meerschweinchenembryo nicht vertragen wird. Eine Menge von Embryonen ging an dieser Behandlung zugrunde […]. Die Gewißheit, daß für den letalen Ausgang der Follikulinbehandlung die Intoleranz des Säugetierembryos gegenüber diesem Stoff und nicht mein operativer Eingriff verantwortlich war, erhielt ich gleich bei den ersten Versuchen, den Meerschweinchenembryo mit männlichem Hormon zu behandeln: Ein jeder blieb dabei am Leben. (Dantschakoff 1941: 25)

Künstlich verabreichtes Follikulin, so konstatierte Dantschakoff in dieser Monografie – und wiederholte das auch an mehreren Stellen in ihren wissenschaftlichen Fachartikeln –, hatte in der „kritischen Phase“ ihrer ersten geschlechtlichen Differenzierung eine tödliche Wirkung auf Embryonen von Meerschweinchen und konnte lediglich in späten Stadien der Embryonalentwicklung appliziert werden, wenn die natürliche Geschlechtsdifferenzierung bereits zu weit fortgeschritten war, um noch weitgehend beeinflusst zu werden (Dantschakoff 1938: 149; 1941: 25). Welche Schlüsse zog die Embryologin aus dieser Beobachtung und welche Konsequenzen könnte dies für den weiteren Verlauf der Entwicklungen auf dem Gebiet der embryonalen Geschlechtsentwicklungsforschung gehabt haben?

In Dantschakoffs Diskussion der Forschungsergebnisse im Jahr 1941 zeichnet sich deutlich ab, dass die konkrete Ursache für die Letalität bei künstlicher Applikation von Östrogenen keineswegs geklärt war und viele andere Szenarien ebenso naheliegend waren – wie die Annahme, dass die weibliche Geschlechtsdifferenzierung ohne die Wirkungen von Östrogenen ablief. „Erfolgt der Tod des Embryos primär oder sekundär? Führt vielleicht ein Mangel an korreliertem Hormon bei der Mutter zum Abort des Embryos? Oder erliegt der Embryo einer direkten Wirkung des Hormons auf seine Gewebe? Und weshalb? Erzeugt doch der genetisch weibliche Embryo selbst in späteren Stadien das Follikulin“, so Dantschakoffs Auflistung der vielen noch offenen Fragen, von denen folgende besonders bemerkenswert ist: „Und zur Zeit der Behandlung bestimmt doch im genetisch weiblichen Embryo der ‚weibliche Determinismus‘ seine weiblich orientiert geschlechtliche Histogenese. Und wodurch?“ (Dantschakoff 1938: 150). Östrogene, so wird hier deutlich, erwiesen sich bei Experimenten mit Säugetieren als widerspenstige „technische Dinge“ der embryologischen Geschlechtsentwicklungsforschung der 1930er Jahre – einer Forschung, die sich in Folge zunehmend auf diese Modellorganismen konzentrierte. Zugleich schließt sich an dieser Stelle der Kreis zu den bereits detailliert dargestellten mikrochirurgischen Kastrationsexperimenten Alfred Josts in den 1940er Jahren sowie zu der eingangs aufgegriffenen Frage: „If Hormones Make the Man, What Makes the Woman?“. Als Josts Untersuchungen an Hasenembryonen suggerierten, alle Versuchstiere würden in Abwesenheit von Geschlechtshormondrüsen wenn auch unvollkommene, aber doch den weiblichen ähnelnde Geschlechtsorgane entwickeln, lag es nahe, den bis zu diesem Zeitpunkt ohnehin nicht ergiebigen Östrogen-Forschungspfad der weiblichen Geschlechtsdifferenzierung zu verlassen und sich auf die Erforschung der Mechanismen der Androgen-vermittelten männlichen Differenzierung in Säugetieren zu konzentrieren. Auch wenn die feministisch-kritische Lesart – die in der hormonellen Abwesenheitshypothese eine Fortschreibung kulturell tradierter Passivitätszuschreibungen an Weiblichkeit erkennt – eine zentrale theoretische Einsicht liefert, verweist die lang anhaltende Persistenz dieser Erklärung auf tiefere strukturelle und epistemische Ausschlüsse innerhalb der Wissenschaftspraxis selbst. Der Blick auf die frühesten Experimentalsysteme der embryonalen Geschlechtsentwicklungsforschung macht darüber hinaus eine historisch weit zurückreichende Pfandabhängigkeit innerhalb der materiell-technischen Bedingungen dieser Systeme sichtbar.^19^

## Fazit

Der Beitrag knüpft an die seit über vier Jahrzehnten formulierte feministisch informierte Kritik an der hormonellen Abwesenheitshypothese weiblicher Geschlechtsentwicklung und dem damit verbundenen Forschungsdesiderat im Bereich der Embryologie an. Während diese Kritik primär die sprachliche Theoriebildung und Themenwahl unter dem Einfluss eines historisch gewachsenen Androzentrismus im biologisch-medizinischen Geschlechterwissen adressiert, richtet die vorliegende Analyse den Fokus auf die materiell-technischen Bedingungen der frühesten endokrinologischen Experimentalsysteme zu Beginn des 20. Jahrhunderts. Er erweitert damit die Perspektive von einigen wissenschaftshistorischen Studien, die bereits die Rolle von Experimenten in Zusammenhang mit der Geschlechtsentwicklung betrachteten, die tierexperimentellen Systeme der endokrinologischen Geschlechtsentwicklungsforschung vor den 1940er Jahren jedoch noch nicht systematisch untersuchten. Es wird gezeigt, dass diese Systeme sich bereits sehr früh als produktive Orte der Erkenntnisgewinnung über die männliche, jedoch nicht über die weibliche Geschlechtsentwicklung erwiesen. Die als „männlich“ konzipierten Keimdrüsenhormone – Androgene – wurden dabei zunächst zu „epistemischen“, später zu „technischen Dingen“ und zentralen Werkzeugen dieser Forschung. Während die ersten beiden Experimentalsysteme – etwa in der Freemartin-Forschung – noch anatomisch-morphologisch arbeiteten, operierten Eugen Steinach und Vera Dantschakoff mit physiologischen und chirurgischen Eingriffen im Modus einer aktiven, intervenierenden Wissenschaft. In diesen Experimenten avancierten zwar auch „weibliche“ Geschlechtshormone – die Östrogene – zu „epistemischen“ und „technischen Dingen“. Sie zeigten sich jedoch als widerspenstige Agentien, die sich der experimentellen Operationalisierung der weiblichen embryonalen Geschlechtsentwicklung im Säugetiermodell weitgehend entzogen. Das daraus resultierende Nichtwissen wurde zunächst noch als Solches reflektiert, bald jedoch durch die Formulierung einer Ex-negativo-Hypothese der *Abwesenheit* von Hormonwirkungen überschrieben. Auf den ersten Blick eine neutrale Schlussfolgerung auf Basis von experimentellen Beobachtungen, hatte dies folgenreiche epistemische Konsequenzen: Zum einen transformierte sich die Beschreibung der weiblichen Geschlechtsdifferenzierung als Prozess, der sich in Abwesenheit von Hormonen vollzieht, allmählich zu einer Konzeption dieses Prozesses als „automatisch“ und „passiv“. Dies geschah, obwohl in Publikationen der ersten Hälfte des 20. Jahrhunderts noch artikuliert wurde, dass die weibliche embryonale Geschlechtsdifferenzierung – wie jeder physiologische Prozess – ebenfalls von spezifischen Faktoren vermittelt sein musste und dass auch eine Rolle der Ovarien in diesem Prozess nicht ausgeschlossen werden konnte. Zum anderen wurden die gut funktionierenden endokrinologischen Experimentalsysteme, die sich der Erforschung der männlichen Geschlechtsdifferenzierung widmeten, immer weiter verfeinert und avancierten zu „Experimentalmaschinerien“ (Rheinberger 2001: 9), die das Wissensobjekt „männliche Geschlechtsdifferenzierung“ durch das anwachsende Wissen über ihre physiologischen Faktoren ebenso stabilisierten wie sie die Frage nach der experimentell (noch) nicht operationalisierten weiblichen Geschlechtsdifferenzierung in den Hintergrund drängten. Damit manifestierten sich Argumentations- und Forschungspfade in der Geschlechtsentwicklungsforschung, die in einem deutlichen Androgen-Zentrismus in diesem Bereich resultierten, wie wir ihn bis in die Gegenwart vorfinden. Die einzelnen forschungsleitenden Entscheidungen – kontroverse Punkte ebenso wie Bereiche des Nichtwissens – wurden allmählich unsichtbar und verschwanden in der Blackbox der Geschlechtsentwicklungsforschung, die mit der vorliegenden Untersuchung zumindest einen Spalt weit geöffnet wurde.

## Footnotes

1 Zur Bedeutung von Geschichten in der Wissenschaft siehe Haraway (1995).

2 Als Androgene oder auch „männliche“ Sexualhormone werden hormonelle Substanzen bezeichnet, die allem voran in den Hoden produziert werden und deren Wirkungen insbesondere mit der Ausbildung spezifisch männlicher Merkmale assoziiert werden. Zu den Androgenen zählen beispielsweise das Hormon Testosteron, Dihydrotestosteron und andere Steroidhormone (Doenecke et al. 2005: 527–529).

3 Seit wenigen Jahren werden die ersten noch vagen Thesen einer nicht-binären Genexpression bei Besonderheiten der Geschlechtsentwicklung und somit einer „molekulare[n] Intersexualität“ diskutiert; siehe etwa Holterhus & Hiort (2021: 379). Das Gros der internationalen Forschungsvorhaben rund um das Thema Geschlechtsdifferenzierung bewegt sich jedoch weiterhin im Rahmen der binär strukturierten und Androgen-zentrierten Forschungstradition.

4 Siehe dazu auch Rheinberger (2001) sowie Rheinberger & Hagner (2018). Eine Überblicksdarstellung von aktuellen Positionen der historischen Epistemologie bietet Vagelli (2024).

5 Diese Technik ist ausführlich dargestellt in Jost (1947b).

6 Aufschluss darüber geben zum Beispiel erste und gleich sehr umfangreiche Handbücher, die sich speziell um die Wirkungen der Androgene drehen – so zum Beispiel Voss & Oertel (1973). Entsprechende und dabei weiniger umfangreiche Übersichtswerke für Östrogene findet man erst seit Ende der 1990er Jahre; siehe etwa Oettel & Schillinger (1999).

7 Siehe dazu ausführlich in Fausto-Sterling (2000: 202; 346; 348).

8 Für eine Überblicksdarstellung und zahlreiche Literaturverweise siehe Voß (2010: 270–276). Für aktuellere Studien siehe das Review von She & Yang (2017).

9 Ausführlich zum Disput über die embryonale Geschlechtsanlage im 19. Jahrhundert siehe Klöppel (2010: 244–259) sowie Voß (2010: 154–158).

10 Siehe dazu auch Honegger (1991), Stoff (2004), Oudshoorn (1994) und Schiebinger (1993).

11 Die Lehre von der „Inneren Sekretion“ wurde erst seit Beginn des 20. Jahrhunderts als „Hormonlehre“ oder auch „Endokrinologie“ bezeichnet. Der Begriff „Hormon“ geht zurück auf den britischen Physiologen Ernest Starling und das Jahr 1905; siehe Starling (1905). „Endokrinologie“ war ein Begriff, den der italienische Physiologe Nicola Pende im Jahr 1912 prägte (Vague 2000: 2688). Für eine umfassende Darstellung der Geschichte der Endokrinologie aus fachinterner Perspektive siehe Medvei (1982). Für historische Arbeiten zur Geschichte der Sexualhormone siehe insbesondere Oudshoorn (1994), Ratmoko (2010), Roberts (2007), Sengoopta (2006) und Stoff (2004).

12 Zur Forschung von Pol Bouin und Paul Ancel siehe Grignon (2000).

13 Das Motiv des „Naturexperiments“ taucht historisch nicht nur im Kontext des Freemartinismus auf, sondern insbesondere auch in medizinischen Diskursen um intergeschlechtliche Menschen ebenso wie um Unfruchtbarkeit. Siehe dazu Klöppel (2006) und Stoff (2004: 419).

14 Die Frage danach, ob die in embryonalen Stadien wirkenden hormonellen Substanzen identisch mit den Hormonen waren, die in späteren Lebensphasen von Organismen produziert wurden, war noch bis in die 1950er Jahre umstritten (Jost 1947a: 163–164).

15 Zitiert nach Stoff (2023: 125). Hier ausführlich zur Etablierung des manipulativ-experimentellen Modus in der Physiologie und Endokrinologie im ausgehenden 19. und frühen 20. Jahrhundert auf S. 123–128.

16 Zu Steinachs Antagonismusthese und zur damaligen Kritik daran siehe Stoff (2004: 439–441).

17 Zu den wichtigen Akteur:innen dieser Forschung zählten zu dieser Zeit neben Vera Dantschakoff beispielsweise der US-Amerikaner Edgar Allen, die Franzosen Jacques Benoit, Robert Courrier und Étienne Wolff sowie der Schweizer Emil Witschi, was sich aus den Publikationsverzeichnissen zum Thema endokrinologische Embryologie oder etwa aus Tagungsberichten entnehmen lässt; siehe Wolff (1951). Eine umfassende wissenschaftshistorische Darstellung der embryologischen Geschlechtsentwicklungsforschung steht bislang allerdings aus.

18 Je nach Publikationssprache findet man unterschiedliche Schreibweisen ihres Vor- und Nachnamens – darunter Véra, Wera oder Vera und Dantchakoff, Danchakoff oder Dantschakoff in verschiedenen Kombinationen. Mit Ausnahme ihrer Erwähnung als „zu ihrer Zeit vermutlich wichtigste Person, die den Zusammenhang von Hormonen (im heutigen Verständnis) und der Entwicklung der Geschlechtsmerkmale experimentell untersuchte“ durch Helga Satzinger (2009: 231–232) findet man nur wenige, überwiegend russischsprachige Artikel über das Leben und die Forschung Dantschakoffs; siehe etwa Fando (2020), Rusakova et al. (2020), Rossiianov (2025) oder Zubritskiy et al. (2024). Die erste umfassende wissenschaftshistorische Aufarbeitung ihrer Biografie ist aktuell Gegenstand eines Projekts der Autorin.

19 Spätestens an dieser Stelle sei angemerkt, dass es neben endokrinologischen Experimentalsystemen und hormonellen Erklärungsmodellen zeitgleich auch andere Forschungsansätze und daraus hervorgehende konkurrierende Geschlechtermodelle gab. Allem voran seien hier die erbanalytische Erforschung der Geschlechtsentwicklung des deutschen Biologen Richard Goldschmidt erwähnt. Siehe dazu ausführlich in Satzinger (2004; 2012).
